# Lateral supraorbital approach for large olfactory groove meningioma and unruptured large anterior communicating artery aneurysm: operative video and technical considerations

**DOI:** 10.3171/2025.10.FOCVID25165

**Published:** 2026-01-01

**Authors:** Nobuyuki Watanabe, Verónica de los Santos, Tingting Jiang, Arianna Fava, Mamoune El Mostarchid, Sébastien Froelich

**Affiliations:** 1 Department of Neurosurgery, Lariboisière Hospital, Assistance Publique–Hôpitaux de Paris; and; 2 University of Paris, France

**Keywords:** lateral supraorbital approach, olfactory groove meningioma, pial infiltration, anterior communicating artery aneurysm, superiorly projecting, single-stage surgery

## Abstract

Anterior skull base meningiomas can be approached via frontotemporal, interhemispheric, lateral supraorbital (LSO), transeyebrow supraorbital, or endonasal transsphenoidal approaches. The LSO approach, with extensive orbital roof drilling, provides a sufficient corridor to reach tumors located most anteriorly while achieving a favorable cosmetic outcome. This video demonstrates the utility of the LSO approach for resection of a large olfactory groove meningioma infiltrating the pia mater and clipping of a large anterior communicating artery (Acom) aneurysm. Even for a superiorly projecting Acom aneurysm, the space created after tumor resection allowed wide interhemispheric dissection and adequate exposure of the aneurysm.

The video can be found here: https://stream.cadmore.media/r10.3171/2025.10.FOCVID25165

## Transcript

This surgical video demonstrates the minimally invasive techniques using lateral supraorbital approach for resection of a large olfactory groove meningioma infiltrating the pia mater with prominent perifocal edema as well as clipping of a superiorly projecting unruptured large anterior communicating artery (Acom) aneurysm.

### 0:41 Clinical History.

A 32-year-old man presented with several months of depression and decreased olfaction in the left nostril. No other neurological symptoms were reported. MRI revealed a 43-mm olfactory groove meningioma with prominent bilateral frontal lobe perifocal edema. Simultaneously, a 10-mm superiorly projecting Acom aneurysm was also identified.

### 1:05 Rationale for Procedure and Surgical Approach.

The rationale for tumor resection was its large size and the associated edema, which caused behavioral and cognitive changes consistent with frontal lobe syndrome. The aneurysm had a wide neck, and because simultaneous treatment with tumor resection appeared feasible, clipping was selected. Several surgical approaches were considered. The classic frontotemporal approach is the most common but requires extensive soft tissue dissection, and the interhemispheric approach provides the most direct access to this type of meningioma and superiorly projecting Acom aneurysms; however, it carries a risk of bilateral olfactory nerve injury. The transeyebrow supraorbital approach is minimally invasive, but the disadvantage is its acute angle to the anterior tumor margin.

In contrast, the lateral supraorbital (LSO) approach offers a sufficient corridor for anterior skull base meningiomas and Acom aneurysms,^1–3^ with improved cosmetic results and potential preservation of contralateral olfactory function. The temporal muscle is detached only at its anterior and superior attachments—along the lateral orbital rim and superior temporal line—without incising the muscle, thereby minimizing postoperative swelling.

A limitation of the LSO approach is the anatomical step between the cribriform plate and the orbital roof, requiring extensive orbital roof drilling. Another drawback is the potential for insufficient exposure of superiorly projecting Acom aneurysms.^4,5^ Nevertheless, the original position and projection of the aneurysm may have been displaced by the tumor. We anticipated that the space created after tumor resection would allow wide dissection of the interhemispheric fissure and adequate exposure of the aneurysm.

### 2:47 Patient Positioning and Skin Incision.

The patient was positioned supine with the head fixed in Mayfield holder, rotated 15° to the contralateral side. A curvilinear skin incision was made just behind the hairline from the 3 cm lateral to the midline toward the left ear up to 3 cm above the root of the zygoma.

### 3:06 Surgical Procedure.

After the skin incision and anterior elevation of the skin flap, interfascial dissection was made, following the deep temporal fascia up to the lateral orbital rim. The pericranium was gently elevated. The temporal muscle was separated at its anterior and superior attachments. A semilunar-shaped bone flap was fashioned with the keyhole at the center of the inferior margin. The anteroinferior margin was extended 3 cm from the keyhole. After the craniotomy, the orbital roof was sufficiently flattened to eliminate the anatomical step toward the cribriform plate and to facilitate access to the tumor attachment.

### 3:51 Intradural Steps and Tumor Resection.

Following a short dural incision, due to brain swelling, CSF was promptly released via the sylvian fissure. The meningioma attachment was immediately reached, and detachment from the dura with devascularization was performed. Devascularization of the dura over the hyperostotic bone surrounding the cribriform plate was completed, primarily targeting the anterior and posterior ethmoidal arteries, followed by the contralateral side.

Comprehensive internal debulking of the tumor was thoroughly performed. The dissection between the tumor capsule and the pia or arachnoid, as we usually perform, was attempted, but disruption of the pia was observed. Therefore, the resection plane was modified to preserve pia mater infiltrated by the tumor.^6^ The interface between the infiltrated pia mater and tumor was identified and carefully dissected with micro forceps in both hands. This represents the dissection plane along the infiltrated pia mater.

Next, the falx was sectioned to expose the anterior portion of the contralateral tumor. A lateral view through the LSO approach provided excellent visualization of the falx. Tumor resection was continued by carefully following the same interface, enabling safe resection and brain preservation. Residual tumor on the contralateral cribriform plate was addressed by drilling the hyperostotic crista galli, which allowed access to this area where the olfactory nerve was identified and preserved. Final view of the surgical cavity appreciating the complete resection of the tumor.

### 5:47 Clipping for Acom Aneurysm.

With tumor resection completed, we moved to aneurysm clipping. The proximal left A1 was identified, and the frontal lobe was elevated from the optic nerve and chiasma. The adequate space obtained after tumor resection eliminated the need for frontal lobe retraction and facilitated wide dissection of the interhemispheric fissure, allowing visualization of the distal left A1, Acom, and the aneurysm. The distal left A1 and both A2 segments were confirmed, and then we started to dissect carefully around the distal neck and proximal neck of the aneurysm.

A temporary clip was placed on the left A1. Because the right A2 was overlying the aneurysm, a fenestrated clip was chosen to preserve flow in the right A2. The aneurysm was clipped with a straight fenestrated clip, while ensuring preservation of flow in the right A2 and confirming the absence of hypothalamic artery around the neck. The A1 occlusion time was 2 minutes. The clip tips were confirmed to extend beyond the aneurysm neck. ICG angiography confirmed complete aneurysm obliteration and preservation of normal blood flow.

After dural closure and bone reconstruction with titanium plates, the fascia and pericranium were closed.

### 7:13 Postoperative Course.

Postoperatively, the patient had no new neurological deficits. Left olfactory function was preserved, and his depression gradually improved. No temporal muscle swelling was observed. Histopathological examination confirmed a meningothelial meningioma, WHO grade 1. Postoperative MRI demonstrated contrast enhancement along the brain surface; however, complete tumor resection was confirmed. No ischemic changes or worsening edema were observed. CT angiography showed complete occlusion of the aneurysm with no remnant, and normal flow, including that of the recurrent artery, was preserved.

### 7:50 Conclusion.

This case demonstrates that a minimally invasive approach can be used to simultaneously treat two complex pathologies. We highlighted the excellent access through the LSO approach to anterior skull base structures, including the cribriform plate and the falx, and described techniques to minimize brain injury when the tumor infiltrates the pia mater. Additionally, wide opening of the interhemispheric fissure facilitated management of a superiorly projecting Acom aneurysm.

## Disclosures

The authors report no conflict of interest concerning the materials or methods used in this study or the findings specified in this publication.

## Author Contributions

Primary surgeon: Froelich. Assistant surgeon: Watanabe, El Mostarchid. Editing and drafting the video and abstract: Watanabe, de los Santos, Fava. Critically revising the work: de los Santos, Fava, Froelich. Reviewed submitted version of the work: Jiang, Fava, Froelich. Approved the final version of the work on behalf of all authors: Watanabe. Supervision: Froelich.

## Supplemental Information

### Patient Informed Consent

The necessary patient informed consent was obtained in this study.
